# Understanding the factors affecting the quantity and composition of street litter: Implication for management practices

**DOI:** 10.1016/j.heliyon.2023.e14245

**Published:** 2023-03-15

**Authors:** Alessandra Rossi, Meiyin Wu, Bernabas T. Wolde, Kevin W. Zerbe, Tsung-Ta David Hsu, Ariane Giudicelli, Rosana Da Silva

**Affiliations:** a Montclair State University, 1 Normal Ave., Montclair, NJ, 07043, USA; b New York-New Jersey Harbor & Estuary Program, 17 Battery Place, New York, 10004, USA

**Keywords:** Floatable debris, Litter management, Stepwise linear regression, Street litter, Socio-economic

## Abstract

Some urban areas have more litter than others. Understanding the reason for this is important not only for dealing with urban littering but also for marine water quality because approximately 80% of the world's marine litter originates on land. This study aimed to better understand the quality and quantity of litter on sidewalks along with the sampling site's socio-economic attributes to better discern why some areas have more/different litter than others and what, if any, are the implications for a more tailored waste management strategy. We surveyed twice each of the 35 sites we selected from the Lower Passaic River watershed and the related Harbor Estuary within New Jersey, U.S.A. A total of 28,431 litter items were recorded with a total mass and volume of 245.8 kg and 4.7 m^3^, respectively. Floatable items accounted for 66% of all objects collected. Cigarette butts were the most numerous among all items (28%) and represented 43% of the total floatable items, the remaining 57% being represented by potentially recyclable items such as plastic, rubber, and Styrofoam. Stepwise linear regression was used to explore the relationship between the litter collected and various predictors. Among others, the results suggest the importance of strategically placing collection bins around properties with relatively lower assessed values, outdoor smoking areas, close to schools, and places where people predominantly walk to their destination. Possible management strategies include prohibiting single use plastic bags, limiting foam food ware, public education, and outreach.

## 1 Introduction

Some urban neighborhoods experience more litter disposal problem than others. This litter can accumulate on the street by overflowing from trash bins and by direct littering. This poses a problem not only for the immediate urban area but also for nearby waterbodies as this litter is blown by wind or washed down to the nearest waterway by surface runoff. Precipitation may also lead to a first flush event wherein microplastics that have accumulated on land during dry periods are flushed into freshwater environments [1,2]. Recent studies have also found that the estimated plastic waste entering the ocean is one to three orders of magnitude greater than the previously reported mass of floating plastic debris in high-concentration ocean gyres. Three quarters of these emissions, which are influenced by anthropogenic and environmental factors such as land cover, slope, and elevation, occur between May and October 1000 rivers contributing to 80% of the global annual emissions [[2], [3], [4], [5], [6]].

Thus, our ability to understand how urban solid waste collection systems work, why they fail, and what can be done to address that problem, has implications for reducing not only street litter and increasing rates of recycling but also for reducing its deposition in local waterways. This is important because single-use disposable plastic products, coupled with their long persistency in the environment, result in the ever-increasing number of plastic objects found in both freshwater and marine environments [7,8].

Moreover, litter has a negative aesthetic impact and it also threatens human and animal safety [9]. Animals can remain trapped in wires and plastic items. Sharp and spiky objects can harm people and other biota and damage valuables [10,11]. In areas where the economy relies on tourism, litter can also reduce revenues [12,13], increase maintenance expenses due to cleaning operations, and cause considerable economic cost on vessels, and fishing gears [10,13,14].

Our street litter project addresses several gaps found in previous studies. Some studies, for instance, used questionnaires [15,16]. Questionnaires provide opinions by the survey takers and can be valuable in gauging awareness, motivation, and perceptions. However, research based on actual, observed litter provides more reliable information on the amount and nature of the litter generated.

The generation of plastic-type wastes depends considerably on socio-economic factors, some of which are not considered in such studies [17]. If the distribution of litter across different areas is dissimilar and there are corresponding differences across these sites in terms of socio-economic attributes as well as the nature of the built environment, among others, it is possible that these differences across the sites help to explain the variation of litter that is observed across the sites. Identifying these factors and the nature or mechanism of their influence on street litter is important to better understanding the existing pattern of litter distribution and what it would take to solve the problem.

Another challenge in the existing literature deals with how the data collection is not standardized by area or per person-hour, making a comparison of results difficult. For example, most surveys in the literature quantified types of litter using counts only [[18], [19], [20], [21]], and did not quantify trash also in volume or mass. The majority of these publicly available reports did not count cigarette butts, which gets washed from the street into a waterbody where it sinks to the bottom after a few days but still poses considerable environmental pollution risks given its degradation-resistance nature and tendency to leak toxic chemicals [18,20,[22], [23], [24], [25]]. Some projects also did not include detailed methodology on how they collected the litter, which made data comparison challenging.

In this study, we conducted street litter collection in communities along a 17-mile tidal stretch that flows through the most urbanized and industrialized area in New Jersey.

The novelty of this study is that we used three different but interrelated approaches, including count, mass, and volume, for measuring litter instead of using just one or two, which provides a less complete picture of the problem being studied. If, for instance, the factors that predict litter by volume do not also predict litter by mass or by count, then it would show that the way we measure litter affects what is learned about the problem and what solutions are developed. One large object being dumped on the street, if measured by volume, can be considered as being the same amount of litter as several hundred smaller sized objects being dumped on the street. However, these two types of litter represent different levels of pollution. Their chances of being carried by a rain event are also different, and so their potential for ending up in the local waterways and in the ocean is different. By using count, volume, and mass as a way of measuring litter, one can get a better sense of the type of litter problem that exists and proceed to develop a more appropriate course of action. From previous studies, we synthesized and developed a survey protocol for collecting and measuring street litter. This protocol is more comprehensive than previous protocols and it also details how to measure the street litter collected. This manuscript thus showcases this protocol and uses the results to explore the nature and cause of the variation in street litter across a study area. We also describe a set of litter management strategies that could be used to address the litter problem that is observed. These strategies mainly focus on reducing the generation of litter encouraging its proper disposal after it has been produced.

The objectives of this study were to quantify and categorize street litter. The study further investigated socio-economic variables that might significantly affect the amount (number, mass, and volume) of litter accumulated along pedestrian sidewalks and streets with goals to prioritize actions for street litter reduction. This information is important in informing management practices that aim to reduce the amount of litter that reaches the rivers, streams, and ultimately the sea. These objectives touch on the United Nations sustainable development goals of Clean Water and Sanitation, Sustainable Cities and Communities, and Life Below Water [26]. By better understanding an addressing these challenges, we can contribute to a more sustainable development.

## 2 Materials and methods

### 2.1 Study area

The Lower Passaic River, the study area, is one of the most polluted areas in the USA, and its basin houses communities with vast differences in their ethnic, educational, and economic patterns [27]. This, together with the high level of urbanization and the large amounts of impervious surfaces, has resulted in both water and sediment contamination, and since 1984 the Lower Passaic River has been identified as a Superfund site and placed on the National Priorities List by the USEPA [28]. Although the extensive industrialization along the waterfront and the commercial shipping have declined in recent years, water quality remains a concern in this stretch of the river.

Various measures, however, are in place to reduce floatable debris along the Lower Passaic River. In addition to the adoption (1988) of the Annex V of the International Convention for the Prevention of Pollution from Ships [29], other programs have been implemented to address the accumulation of litter, including the Marine Protection, Research and Sanctuaries Act [30], also known as the Ocean Dumping Act, and the Marine Plastic Pollution Research and Control Act [31], which are especially relevant to the tidal areas and navigable waters. In 1989, collaboration between the neighboring two states (New Jersey and New York) and several federal agencies, including the USEPA, generated a Floatable Action Plan [32].

This plan currently includes floating barriers or booms that contain debris for later removal, skim vessels that collect the floatables, street sweeping, adopt-a-basket, clean-streets programs, and clean-beaches programs, among many others. Despite all the efforts, the litter problems on the shorelines of the two states still persist. Food and drink-related litter is still a persistent problem, 47% coming from food wrappers, bottle caps, lids, beverage bottles and cans, straws and stirrers [33]. The balance is composed of cigarette and cigar butts, foam and plastic pieces, and plastic bags, among others [33]. To address this problem, as of January 2020, at least 34 municipalities and two counties in NJ have made effective plastic bag regulations, and eleven municipalities are waiting for some rules to go into effect [34]. Moreover, the state of New York and other seven states (California, Maine, Hawaii, Connecticut, Delaware, Oregon, and Vermont) have put a ban on single-use plastic bags at large retail stores [35]. Despite these measures, floatables remain one of the main threats to water quality in the area [36]. The municipalities near the study area in the Hudson-Raritan Estuary watershed alone invest almost $60 million annually on activities involving preventing marine debris [37].

**Fig. 1:**
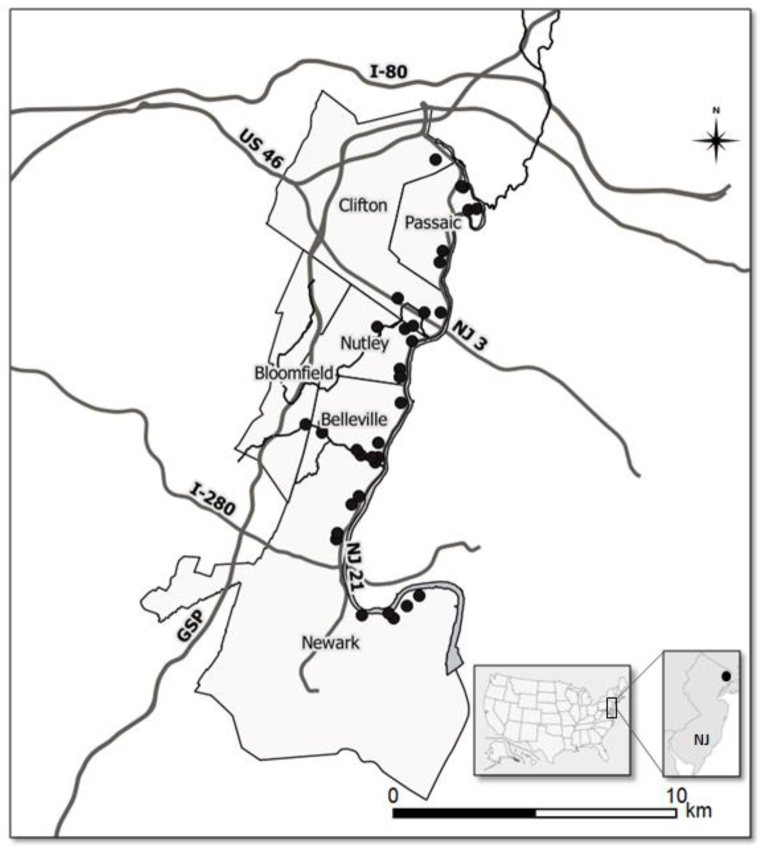
Map of the investigated area. The solid black dots show the individual sampling sites along the Lower Passaic River, Second River, and Third River. Major highways and the townships or cities involved are also indicated (I-80, I-280, US 46, NJ 21, NJ 3, and Garden State Parkway (GSP)).

In addition to the Lower Passaic River, in this study we investigated streets along two of its tributaries, the Second and Third rivers, which cross several towns and cities before joining the Passaic River. Fig. 1 shows the map of the study area along with the sampling sites that were investigated.

Demographic data for the investigated area were retrieved from the 2016 American Community Survey (ACS) 5-year estimates at the block-level for portions of the municipalities overlapping with the sampling sites. These data included variables such as population density, median household age, and mean per capita income, among others. A block or a census block is the smallest aggregation unit offered by ACS. It is a statistical area delimited by visible and non-visible features, such as streets, roads, streams, and property lines. A city block is bounded on all sides by streets. In the suburbs and rural areas, a block might be bigger, more irregular in shape, and bounded by a variety of features, such as roads, streams, and transmission lines. When average income or other variables are reported for a given area, the smallest area for which that type of census data can be reported is the block level. For a given block, we collected litter from sampling sites, which are 400-m walkway segments. More detailed demographic information is available in Table 1. This table illustrates the high demographic variability of the sampling sites [27].

**Table 1:** Summary of the demographic attributes of general area investigated in the present project. Municipalities include Belleville, Bloomfield, Clifton, Newark, Nutley, and Passaic. For each subcategory listed, average (Mean), minimum (Min), and maximum (Max) values presented, are weighted average values of all the sampling sites. Note: The *Not in labor force* subcategory includes 80.5% of the unemployed (American Community Survey and U.S. Census Bureau, 2016).

Categories	Subcategories	Mean	Min	Max
**School enrollment for the population ≥ 3 years old**	Preschool to High school	123	0	859
	College & up	77	0	387
**Educational attainment for the population ≥ 25 years old**	Preschool to High school	160	0	1825
	College & up	273	0	1407
**Employment status for the population ≥ 16 years old**	Employed	1152	199	3066
	Unemployed + not in labor force (80.5%)	728	104	2054
**Household type by household size**	Average people per household	3	2	4
**Total population in occupied housing units by tenure**	Owner	857	24	4194
	Renter	1564	83	5664
**Value of home (Number of housing units within specific value ranges)**	Value between $0 & $500,000	93	0	980
	Value between $500,000 & $1,000,000	14	0	130
	Value > $1,000,000	0.3	0	12
**Median household income ($)**	Median household income	56,103	15,625	124,650
**Median age by sex (years)**	Male	33.3	21.8	45.2
	Female	34.1	23.6	44.2
**Race**	White alone	1294	174	3396
	Black or African American alone	462	18	2184
	American Indian and Alaska Native alone	9	0	61
	Asian alone	166	0	1119
	Some other race alone	410	0	1982
	Two or more races	110	0	678
**Means of transportation to work**	Car, truck, or van	783	39	1969
	Public transportation (included taxicab)	222	27	977
	Two wheels (motorcycle or bicycle)	3	0	19
	Walked	75	0	334
	Other means of transportation	21	0	175
	Worked at home	26	0	113

### 2.2 Sampling sites selection

We compiled an initial list of 140 sites on the west side of the Lower Passaic River in Essex and Passaic counties through a random selection using QGIS Desktop 2.18. A specified number of random points can be generated inside or along the constraining feature (polygon, line, or point) such as rivers or segments of a river or other watershed features. Points are then randomly placed inside the applicable feature.

We constrained the selection within 300 m from a river channel and within a 3-river-kilometer upstream limit for the two tributaries. The first filter was applied on the map, removing all spots that fell in the middle of the water channel and on highways. We then performed in-person scouting to assess the suitability of the sites selected in terms of representation of the entire area investigated in 2018. Criteria that helped with this selection were: access to the waterway, grade of littering at first glance, presence of a walkway for consistency and safety, and vicinity to parking for operations of loading and unloading the litter collected (more details can be found in the Survey Protocol section in the Supplementary material). Among the final list of 35 sites, featuring residential, commercial, and industrial properties, 24 sites were located along the Lower Passaic River, six along the Second River, and five along the Third River spread across the townships of Passaic, Clifton, Nutley, Bloomfield, Belleville, and Newark, New Jersey (Fig. 1).

### 2.3 Survey protocol

We developed a USEPA-approved quality assurance project plan (QAPP) which included a detailed Survey Protocol for data collection (Supplementary material) addressing the challenges and gaps encountered in previous studies focusing on litter found on streets or sidewalks. We visited each of the 35 sites twice between October and December, which is the main rain season wherein the floatable litter is more likely to be transported and deposited in the local waterbodies by rainfall/runoff events, eventually ending up in the sea. Collecting the data twice allowed us to avoid the risk of errors or randomness associated with a one-time only type of data collection approach [38]. To further improve the measurement of litter, we accounted for the number of days our collection event differed from the trash collection date.

We collected and recorded all litter items equal to or larger than 2.5 cm in length, sorted them based on categories (e.g. food, drinks, household items, among others–the full list and corresponding subcategories are listed in Table 2), and quantified them by count, volume, and mass in accordance with the above mentioned protocol. We avoided collection within 48 h from municipal litter pick-up, street sweeping schedules, and/or a rain event capable of creating any noticeable runoff. We limited every site visit to a 400-m walkable segment on one side of the roadway or street and about 30 cm into the street or until the end of a storm drain (also referred to as a catch basin). The average width of walkways, which are all paved, is 1–1.2 m wide, some being as wide as 1.5 m. Litter that was on the vegetation between the walkway and the street was also collected.

**Table 2:** Summary of categories and subcategories. Glass fragments, syringes, condoms, pet waste bags, and large household items were counted but not collected. For each subcategory listed below, the material out of which the litter was made was also recorded and that included such materials as glass/ceramic, plastic, metal, composite, Styrofoam, paper, aluminum, cardboard, rubber, wood, textile, rock, and organic.

Categories	Subcategories
Drink-related items	Liquor bottles/cans; Non-liquor bottles/cans; Juice/Milk boxes; Cups, straws, lids, caps, ring holders, pull tabs
Food-related items	Candy/snack wrappers; Food wrappers/packaging/containers; Plates/utensils; Ziplock bags
Medical-related items	Drug vials; Condoms; Bandages/wound wrappings; Cotton swabs; Syringe
Organic waste	Human/pet waste; Food waste; Yard/leaves waste
Large & household items	Furniture/mattress; Bags with litter; Tires/wheels; Appliances; Shopping cart; Vehicle battery or parts; Bike/vehicle
Tobacco-related items	Lighter; Cigarette/cigars butts; Tobacco wrap/box; Cigarette holder; Matches
Construction materials/tools	Concrete/bricks/tiles/wood parts; Tools/rebar/gloves
Miscellaneous	Ball/toy; Batteries (non vehicle); Office supplies; Personal/home care/chemicals products; Hose/wire/cable/rope; Wipes/dryer sheets; Non-food wrapping/packaging/containers; Diapers/pads; Grocery/shopping bags; Tags/labels/napkins/tissues; Newspaper/magazine/office paper; Cardboard; Shoe/clothing/bedding; Dead animals
Fragments	Fragments/pieces (all materials)
Other	Specify

Each collector completed a day-long training on the survey protocol prior to any data collection per the QAPP requirements. We prepared a survey form and a tally form *ad hoc* to record detailed information at each site. The forms were designed using information gathered from previous studies [[18], [19], [20], [21],[39], [40], [41]]. Among others, the data included such categories as traffic in 10-minute interval and food/drink related businesses. The traffic data was further divided into vehicle traffic and foot traffic. The food/drink related business data was further divided into grocery stores, restaurants/diners, liquor stores, fast food restaurants, food carts/trucks, convenience stores, and coffee shops.

Table 2 presents a detailed list of items that are grouped into categories (e.g., drink or food-related, tobacco related) and subcategories (e.g., bottle, can, food wrap). We also quantified floatable items, which we are defining as constituting plastic, rubber, Styrofoam, and cigarette butts. Whereas we counted it, we did not physically collect bulk litter (e.g., televisions, mattresses, tires) or objects smaller than 2.5 cm in length (approximately the size of a cigarette butt).

Mass was measured using an *Acculab* weighing scale. The items were bagged and then the bag was placed on the scale. To measure volume, the items were placed in a bag and were compressed. The bag was then placed in a bin with predefined measure of volume. The volume of the litter was deduced based on how much of the bin the litter took up. The litter was then properly disposed.

Along with the distribution of street litter, the socio-economic attributes of the sites are not uniform. We downloaded census block level data on such attributes from the Census Bureau's website. This included income, employment status, household size, property value, and level of education [27]. For 400-m segments that spanned more than one block, the weighted average approach was applied.

We were also able to account for the effect of the built environment such as the presence of schools on litter distribution by using proxies like local resident's school enrollment statistics. We also used proxies including main mode of transportation to gauge the role of transportation infrastructures such as bus stops, train stations, sidewalks, and parking structures. This helped us to account for the quality of neighborhood by using proxies like property value. These variables allow us to consider not only individual factors but also neighborhood, socioeconomic, and infrastructural factors in affecting litter distribution across an area and in determining how best to manage it. Table 3 lists all the parameters.

**Table 3:** Categories and subcategories used as socio-economic predictors for statistical analysis. Data retrieved from U.S. Census Bureau, 2012–2016 American Community Survey 5-Year Estimates (U.S. Census Bureau & ACS 2016).

Parameters	Subcategories: socio economic predictors included in the analysis
**School enrollment by detailed level of school for the population ≥ 3 years old**	Not enrolled in school; Preschool; K-grade 5; Middle school; High school; Enrolled in college (undergraduates); Graduate or professional school
**Education attainment for the population ≥ 25 years old**	No schooling completed; Preschool; K-grade 5; Middle school; 12th grade, NO diploma; High school w & w/o diploma; Some college (NO degree); Professional or college degree; Graduate or professional degree
**Employment status for population ≥ 16 years old**	Total labor force (16 yrs & up); Unemployed + not in labor force
**Total population**	Tot pop
**Household type by household size**	Total households; Average people per household
**Housing units**	Total housing units
**Total population in occupied housing units by tenure**	Owner occupied; Renter occupied
**Value (of home)**	Value < $100,000; Value between $100,000 & $300,000; Value between $300,000 & $500,000; Value between $500,000 & $1,000,000; Value > $1 million
**Median household income**	Median household income in the past 12 months (in 2016 inflation-adjusted $)
**Per capita income**	Per capita income in the past 12 months (in 2016 inflation-adjusted $)
**Median age by sex**	Median age – Male; Median age – Female
**Race**	White alone; Black or African American alone; American Indian and Alaska Native alone; Asian alone; Native Hawaiian and Other Pacific Islander alone; Some other race alone; Two or more races
**Means of transportation to work**	Car, truck, or van (four wheels); Public transportation (included taxicab); Two wheels (motorcycle or bicycle); Walked; Other means of transportation; Worked at home

### 2.4 Statistical analysis

Some sampling sites fall within a census block. Others span more than a given census block. For these sites, we used a weighted average approach by considering how much of the segment fell within the multiple census blocks. We weighted each census block's share of the sample for each sampling site, which enabled a specific input of each census block to the amount of litter collected.

In addition to characterizing the litter collected and providing descriptive statistics of the same, we analyzed the factors that predicted the distribution of litter by applying a stepwise linear regression analysis, using JMP Pro 14 statistical software. We used stepwise regression analysis to select the variables to be included in the final regression model. This approach provides a more objective way of selecting variables for consideration and avoids subjectivity. This also allows us to break variables into statistically distinct subgroups and to study them more closely instead of measuring a single coefficient for that variable.

We treated these socio-economic variables as potential predictors for the litter found at the study sites. Variables such as middle school enrollment and foot traffic were selected because they were reported to affect street litter as documented in previous studies [42,43]. The data for our analysis also came from the Census Bureau. While this has its limitations, it also allows us to ensure consistency across the different sites considered in the study. Some of the factors considered in this analysis were also new to this type of study. This includes factors like the area's make up in terms of school enrollment and employment status. With such factors, we were more interested in seeing if and how they affected street litter rather than us having a predefined notion of how they affect it and seeing if that was confirmed.

We also accounted for other predictors such as the average number of days elapsed between either the scheduled litter pickup or the street sweeping and the day of collection at each segment. We used all three different metrics for the litter collected, count, volume (cm^3^), and mass (g) as the dependent variables in the regression models.

The model for simple linear regression is given by equation [1]

[1] Y = β_0_ + β_1_X_1_ + bβ_2_X_2_ + β_3_X_3_ …..β_n_X_n_ + ε

where **Y** is the dependent variable, β_0_ is the y-intercept, the Xs are the independent variables, β_1_ to β_n_ are the coefficients of the respective independent variables, and **ε** is the random error variable.

## 3 Results and discussion

### 3.1 Breakdown of the litter collected

We recorded a total of 28,431 street litter items as a result of the surveys we conducted throughout the project at the 35 sites, equivalent to a total mass of 245,781 g, and a total volume of 4,737,520 cm^3^. The average count, mass, and volume of items recorded for each sampling site are 812 items, 7022 g, and 135,358 cm^3^, respectively.

Plastic, rubber, and Styrofoam materials accounted for 57% of the number of floatable litter collected. By count, the most frequently encountered litter was cigarette butts, representing 43% of all the buoyant objects collected and 28% of total litter collected. This was comparable to results reported elsewhere, including a statewide survey of Texas in 2005, 2009, and 2013 [21]. These results suggest that strategies aiming to reduce litter should target, among others, cigarette litter by providing outdoor ashtrays close to designated smoking areas. A littering behavior study of 130 outdoor public sites in the United States revealed that 82% of the investigated sites were littered with cigarette butts and that, on an average, 65% of the observed smokers were littering cigarette butts. This behavior likely follows from the restriction of indoor smoking that has led to people smoking outdoors and, in the absence of collection bins, the tossing of cigarette butts on the streets [44]. A previous study [15] showed that 82.4% of the surveyed respondents were not aware or disagreed with the non-biodegradability of cigarette butts and emphasized the need to educate people on basic environmental concepts. This is important because, despite their cotton-like appearance, cigarette butts are primarily made out of compressed, plasticized cellulose acetate. The acetylation process converts degradable cellulose into a degradation-resistant substance called cellulose acetate fibers [22,25]. This waste remains in the environment for a long time. Even in marine environments, the durability of cigarette butts and the leakage of toxic chemicals from them can last up to thirty years [23].

**Fig. 2:**
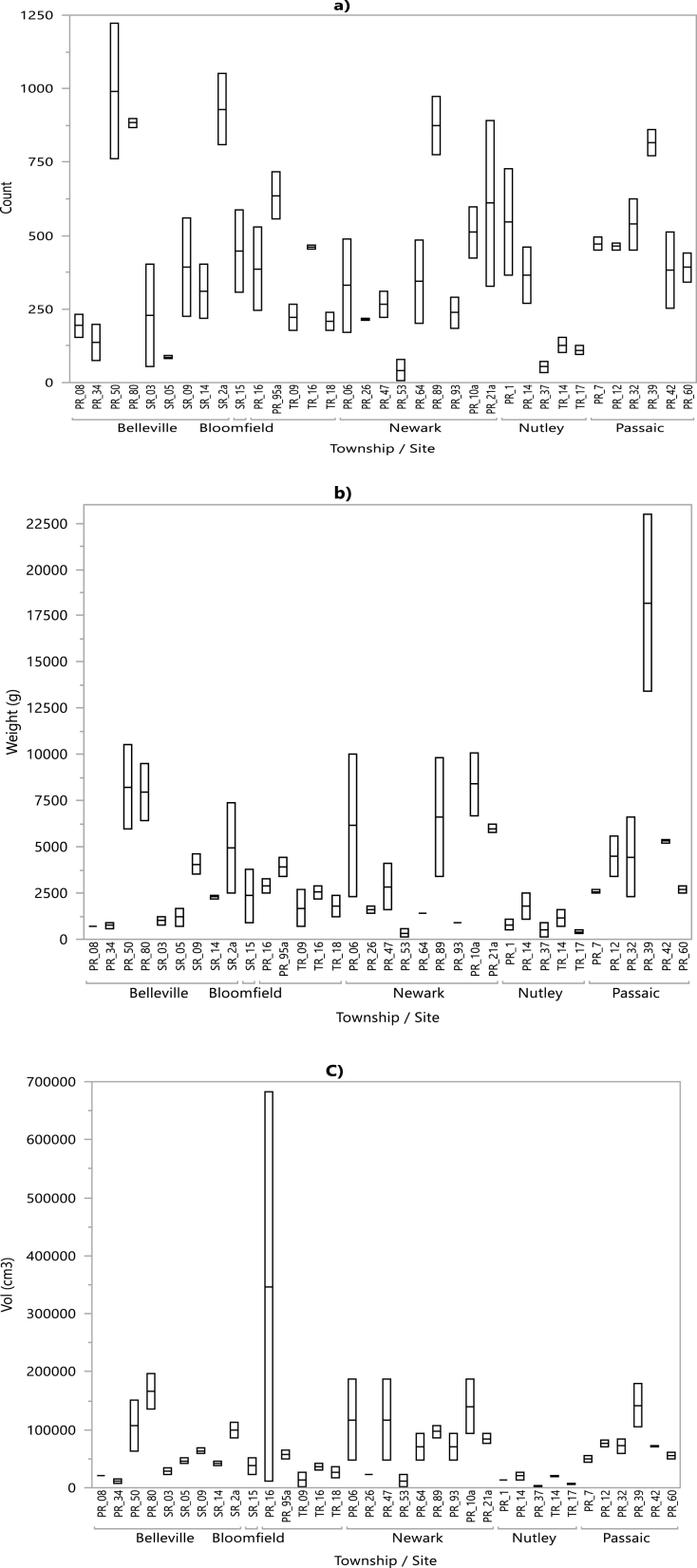
Counts (a), mass (b) and volumes (c) of items recorded per site, arranged by township. Only one site was surveyed in the township of Bloomfield.

Fig. 2(a–c) shows the variation in the amount of litter across the study areas and highlights the importance of using multiple ways of measuring litter. With 1981 items, a site located in Belleville had the largest quantity (in number) of street litter (Fig. 2a), whereas a site in Passaic had the highest mass value of street litter of 36,372 g (Fig. 2b). A site in Clifton had the highest volume of street litter out of all the sites investigated, with a recorded volume of 693,606 cm^3^ (Fig. 2c). Which area is considered to have the biggest litter problem and thus prioritized for management decisions depend on the type of measure used to quantify that litter. Areas that rank low in terms of litter count can rank higher if the litter is measured in either mass or volume. These results revealed the importance of using various ways of measuring street litter in prioritizing litter pickup allocation. Whereas some sites scored high in volume or mass, others did so in number of items collected. Areas with both large count and volume/mass of litter for instance should receive greater attention than areas with just large number of count or volume/mass. Having data on these various measures, thus, allows the responsible parties to make informed decisions in allocating their finite resources.

**Fig. 3:**
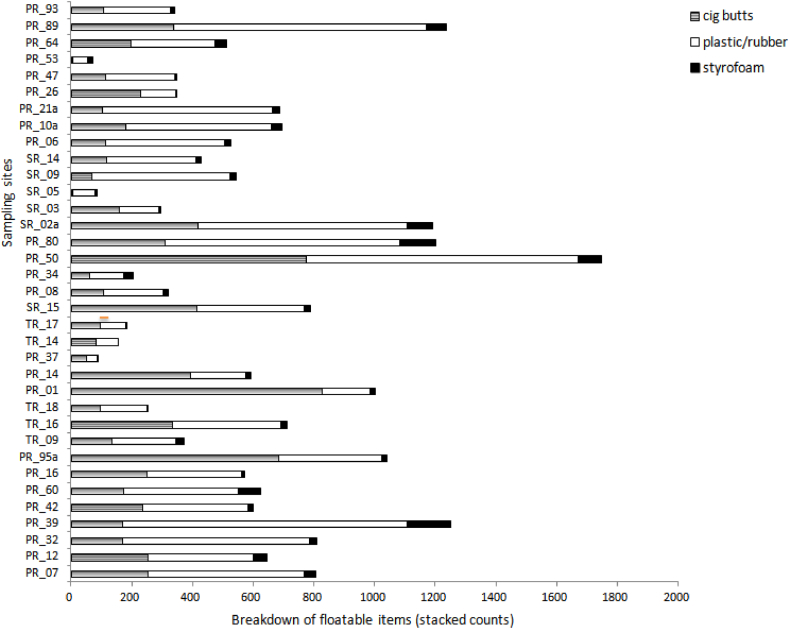
Breakdown of the number of Styrofoam, plastic/rubber, and cigarette butts across the sampling sites.

The results also revealed dissimilarity in the composition of the litter collected at the various sampling sites across all five townships (Fig. 2(a–c)). Although food and drink related items were among the most commonly encountered litter (32% of all the collected items), their average contribution to the total amount of litter collected varied considerably (12%–60% among sampling sites). While floatable objects such as Styrofoam, plastic/rubber, and cigarette butts accounted for 66% of the total number of items collected, this value ranged from 44.3% to 86.8% among study sites and mirrors the breakdown of litter found on beaches [45]. This result further showed the dissimilar distribution of street litter, even across relatively close geographic areas (Fig. 3).

Fig. 3 showed not only that the sampling sites are dissimilar in terms of the floatable litter they feature but also that some sites have more litter than others. This has implication for nearby waters because during flooding and other major precipitation events, both floatable and non-floatable litter can be transported and suspended throughout the water column. As a consequence, rivers near these areas will likely have higher concentration of plastic [5,46]. Although not all of the litter that gets into the river ends up in the ocean, it still represents a pollution concern for the riverine environment. Given that it is less costly to prevent the problem rather than remediating it afterwards, addressing the street litter problem before it enters the water systems is a prudent measure [47].

### 3.2 Predictors of litter distribution

Despite the differences in the way the litter was measured (count, mass, and volume) and the dissimilar distribution of litter (by type) across the sampling sites, results of the regression analyses showed common patterns that can help us identify the best strategies for tackling the street litter problem.

Analysis of foot and vehicle traffic found no correlation between litter volume and vehicle traffic (R^2^ = 0.011) or foot traffic (R^2^ = 0.009). We found a weak correlation between foot traffic and litter mass (R^2^ = 0.282, *p* value of 0.0001) and litter count (R^2^ = 0.119, *p* value of 0.0031). In general, foot traffic seemed to have a greater impact on the amount of litter on the street than vehicle traffic, and it had been observed also in other studies [42,[48], [49], [50], [51], [52]]. The results of stepwise regression analyses revealed the factors that predict, with 95% and 99% levels of confidence, the street litter collected at the investigated sites (Table 4). Demographics of the local residents, for instance, significantly predicted street litter. Sampling sites with a relatively higher number of middle school aged children or younger tended to have relatively fewer and lighter amounts of litter as compared to sampling sites that had predominantly high school aged children (*p < 0.05)* (Table 4)*.* The outcome relative to the *Middle school students* may be explained by the fact that children at this age walk alone less to school because they are more likely to be accompanied by an adult or they take the school bus. They would then have less occasions to litter because of adult supervision or because they spend less time on the streets alone. *High school students* may be more unsupervised and have longer class hours and money for snacks, food or drinks and may be more likely to improperly dispose of the food wrappers and/or drink containers. A previous study [43] has identified both middle and high public schools prone to show higher levels of litter within a buffer distance of about 460 m from the school buildings or the convenience stores/fast food restaurants.

**Table 4:** Parameter estimates for the stepwise linear regression of average counts, average volume analysis, and average mass analysis against all the predictors listed in Table 1 plus street sweeping and litter pickup. Significant variables are indicated by *95% and **99% confidence level. Parameter estimate, Std error, t Ratio, and Prob>|t| are also presented in the table.

	Socio economic predictors	Estimate	Std Error	t Ratio	Prob>|t|
**Average Count analysis**	Middle school (school enrollment)	−1.485	0.836	−1.78	*0.0815*
	No schooling completed	−1.796	1.387	−1.29	*0.2012*
	Total population	−0.176	0.110	−1.59	*0.1172*
	Total housing units	0.837	0.288	2.91	0.0054**
	Median age –Female	1.801	2.238	0.8	*0.4246*
	Asian alone	−0.426	0.214	−2	0.0511*
	Two wheels (motorcycle or bicycle)	−6.518	4.508	−1.45	*0.1543*
	Other means of transportation	2.039	0.956	2.13	0.0378*
	Waste pick up frequency	43.732	23.949	1.83	*0.0736*
**Average Volume analysis (m**^3^**)**	Not enrolled in school	−5.62E − 05	3.52E − 05	−1.6	*0.1166*
	K-grade 5 (school enrollment)	−0.0002	9.25E − 05	−1.7	*0.0964*
	High school (school enrollment)	0.0004	0.0002	2.21	0.0315*
	Graduate or professional degree (education attainment)	0.0003	9.92E − 05	2.93	0.0051**
	Value $100,000 - $300,000	0.0006	0.0001	4.7	<0.0001**
	Value $500,000 - $1,000,000	−0.0009	0.0005	−1.69	*0.0974*
	Median age Female	0.0004	0.0005	0.83	*0.4104*
	Two or more races	0.0004	8.52E − 05	4.27	<0.0001**
	Car, truck, or van (four wheels)	−0.0001	4.74E − 05	−2.37	0.0218*
	Two wheels (motorcycle or bicycle)	0.003	0.001	2.54	0.0145*
	Walked	0.0004	0.0001	2.76	0.0081**
	Other means of transportation	0.0008	0.0002	3.39	0.0014**
**Average Mass analysis (g)**	Middle school (enrollment)	−0.020	0.009	−2.17	0.0338*
	Renter occupied	0.001	0.0006	1.75	*0.0853*
	Median age –Female	0.041	0.014	2.89	0.0055**
	Other means of transportation	0.057	0.012	4.9	<0.0001**

Note: The F statistics for the analyses of variance for the models we ran are 15.4 and 20.43, both having a p values of 0.0001. Given that the F statistic is greater than one and that the corresponding p values are significant, we can tell that the estimated slope parameters are different from zero. The lack of fit test further confirms this result. For F ratios of 1.6 and 0.58, the corresponding p values are 0.17 and 0.99. There is, thus, sufficient evidence at the α = 0.05 level to reject the null hypothesis that there is a lack of fit in the regression model.

Our study also found a positive and statistically significant relationship between the number of food and drink related businesses in an area and the number of food and drink related litter generated in that area. With a *p* value < 0.001 and an R^2^ value of 0.81, our result shows that each additional food and drink related business leads to 10.6 food and drink related litter to be generated on the street it is located on.

Contradicting the common belief that a higher educational level results in a greater awareness of how to properly dispose of litter, the *Graduate and professional degree* educational attainment for people 25 years and older category showed a positive correlation (*p = 0.0051*) with volumes of litter (Table 4). This may be due to a more active social life for people of this age class, more purchasing power, more access to food, drink and cigarettes [51].

Regarding the *Housing-unit* variable, the results show a positive relationship between the total number of housing units and the amount of litter generated (*p = 0.0054,* Table 4). On the other side, the tenant type (*Renter* or *Owner*) and the *Household size* did not show any significant relationship with the abundance of items on the street. A housing-related predictor that seemed to increase the volume of street litter was the presence of homes with a *Value between $100,000 & $300,000* (Table 4, *p < 0.0001*), which is below the median value of owner-occupied housing units in our study area, which is approximately $315,000 [53]. The property taxes from such houses and, by extension, the amount of resources available for upkeep of those neighborhoods, are low.

*Employment status* and *Income* did not have a significant statistical relationship with any of the three methods of litter quantification, although other studies (not involving litter pickup) showed that low-income neighborhoods have more litter [54,55].

There was a positive relationship between *Median age females* and litter mass records (Table 4, *p = 0.0055*). Among the race subcategories, only the *Asian alone* and the *Two or more races* groups showed a significant relationship with litter counts and volumes, respectively. *Asian alone* showed a negative relationship (*p = 0.0511*) while the *Two or more races* predictor shows a positive relationship (*p < 0.0001*). Another research identified the number of Hispanic residents to be positively related with amounts of litter [56] (*p < 0.001*).

Finally, the *Means of Transportation to work* had the highest number of significant predictors addressing the three methods of quantification of litter. The modes of transportation considered in this study included *Car, truck or van* (four wheels), *Two-wheel* (motorcycle or bicycle), *Walked*, *Worked at home*, *Public transportation* (bus or trolley bus, street or trolley car, subway or elevated railroad, ferryboat, and taxicab) and *Other means of* transportation which could include ridesharing activities. In particular, the subcategory Other means of transportation (Table 4) appeared significant at the 95% confidence level for litter counts (*p = 0.0378*), volumes (*p = 0.0014*), and masses (*p < 0001*). The correlations of this subcategory with the three methods of litter quantification revealed that when the option *Other means of transportation* is preferred, the amount of litter deposited on the street is increased. *Other means of transportation* were not described in the ACS-Census Bureau website, but they might refer also to scooter, skateboard, and electric board. Our results also showed that the *Two-wheel* and *Walked* (Table 4) categories had a positive relationship with the volume of litter (*p = 0.0145* and *p = 0.0081,* respectively), while *Car, truck or van* was negatively related to litter volume (*p = 0.0218*). A practical example is given by the column *Estimate* in Table 4 where it shows that for any additional person traveling to work by *Other means* the number of litter items would increase by a factor of two. Another practical example is the contribution given by people in the *Walked* subcategory. The *Estimate* value of 0.0004 for the volume analysis (Table 4) means that for any additional person traveling to work by walking, there is 0.0004 m^3^ (or 400 cm^3^) more litter found on the street. As mentioned earlier, similar findings related to pedestrian traffic and increased amounts of street litter have been observed in other investigations [42,48,49].

In summary, our results showed that priority in the allocation of collection bins should be given to areas with properties with relatively low assessed value, within close proximity to high schools, and neighborhoods where people mostly walk for their daily movements, and around designated smoking areas [43].

### 3.3 Management options

The measure of mass, count, and volume will help decision-makers identify which areas to prioritize for cleaning efforts. Once the priority areas are identified, which litter management measures should be taken will depend on the context and needs of that area, as the solutions that work for one area/watershed might not be applicable/relevant for others. Applicable management options found in the literature include such measures as reducing the use of single use bags. Single use carryout bags have been found to contribute substantially to the litter stream [57]. Applicable control measures for mitigating this problem include a prohibition of such bags at large supermarkets and the prohibition of their distribution at retail establishments that sell packaged foods [58]. Outside of banning such bags, retail establishments can also consider charging a mandatory fee for single-use bags [59]. Food service vendors can also consider providing consumers a discount for bringing their own reusable food and beverage ware [60].

Municipalities can also incentivize such establishments as retailers, supermarkets, and food vendors to limit/prohibit giving out foam food ware and other types of litter by giving them load reduction credits that can be redeemed for some money, service, or to avoid penalties associated with noncompliance [59].

Public education and outreach efforts can also inform local businesses and residents about stormwater issues related to floatable litter, watershed awareness, and pollution prevention. This can be done by developing brochures and other media as well as by engaging in active online campaigns. The outreach can also target school-age children and youth [61]. Such an outreach program can also include a pre and post-campaign evaluation component to determine the effectiveness of that intervention and to determine how to alter/adapt the program going forward [62]. In addition to engaging the public this way, municipalities and other interested parties can also invite the public to participate in a citizen science type of activity where the public can help in collecting and reporting data on floatable litter they see on their streets [13].

Although enforcement of anti-littering laws focuses on larger scale illegal dumping, giving out citations and prosecution for small littering events could also be considered [63].

The municipality can also ensure that there are adequate private trash services especially in areas where businesses and households have inadequate/inefficient trash collection [64]. This is more important in high-trash-generating areas such as parks and near schools.

Where specific types of street litter such as cigarette butts, which are driven by laws restricting indoor smoking, are more likely to be found, installation of specialty trash bins/containers such as outdoor ashtrays is also recommended [44].

Actions that could increase the effectiveness of existing street sweeping practices are enforcing the removal of vehicles from streets during street sweeping to allow sweepers to reach the curb as well as enhancing the frequency of street sweeping in high trash generating areas [59,65].

Finally, municipalities can also consider installing/increasing the maintenance frequency of litter capture devices such as curb inlet screens and litter booms/curtains which block trash from entering a storm drain [66].

A policy action banning single use plastic or imposing cost on buyers for using those bags could face social resistance. Educating the public about the benefits of doing so and making alternative bags affordable will possibly help with this transition. Future studies can explore the best possible ways to make this a reality.

## 4 Conclusions

The results of this study revealed that the way street litter is measured matters in terms of which areas are prioritized for cleaning. Measuring litter in multiple ways thus offers more information in better profiling relevant areas for waste management. The results also showed considerable variation in the type, mass, and volume of street litter distribution across areas. The types of litter that are predominant in one area were not necessarily the same ones that are predominant elsewhere. Given that different litter types result in different environmental costs and require their respective ways of management, it is important to know what types of litter exist and where. This way, litter and the appropriate mode of clean up can be matched for a greater effect.

Moreover, the results showed that, despite the variation in litter distribution, common patterns exist in terms of the factors that help to understand why some areas have more street litter than others. In particular, litter reduction strategies should be focused on reduction of cigarette butts and food and drink related litter, which often originates from convenience stores and fast food restaurants. Socio-economic attributes, including proximity to schools (i.e. middle schools and high schools), property value, designated outdoor smoking areas, and the predominant mode of getting around the neighborhood, significantly predicted street litter. By accounting for these factors decision-makers can better tailor waste management strategies to suit specific areas.

Future studies can assess the correlation between a local marine litter and local street litter. To account for a possible seasonality of litter resulting, for instance, from the diminished likelihood of spending time outdoor during the winter season as compared to the summer season, future studies can compare street litter distribution across the different seasons and determine the required management strategies, especially in temperate areas where inter-season differences of temperatures are noticeable. Such studies can also distinguish between the recyclable/non-recyclable shares of the litter that exist and devise strategies for further improving recycling practices. Moreover, while the results show that enrollment in certain school levels corresponds with greater/lesser incidences of littering, why exactly that is and how it manifests are topics that need to be investigated.

## Author contribution

Alessandra Rossi: conceived and designed the experiments; performed the experiments; analyzed and interpreted the data; contributed reagents, materials, analysis tools or data; wrote the paper.

Meiyin Wu: conceived and designed the experiments; analyzed and interpreted the data; Contributed reagents, materials, analysis tools or data; wrote the paper.

Bernabas T. Wolde: analyzed and interpreted the data; contributed reagents, materials, analysis tools or data; wrote the paper.

Kevin W. Zerbe: performed the experiments; contributed reagents, materials, analysis tools or data.

Tsung-Ta David Hsu: contributed reagents, materials, analysis tools or data.

Ariane Giudicelli: conceived and designed the experiments; contributed reagents, materials, analysis tools or data.

Rosana Da Silva: analyzed and interpreted the data; contributed reagents, materials, analysis tools or data; wrote the paper.

## Funding statement

This work was supported by New England Interstate Water Pollution Control Commission (NEIWPCC) [0323-003].

## Data availability statement

We are using some version of these data along with other primary data to develop another manuscript, at which point we will release all the data.

## Additional information

Supplementary content related to this article has been published online at [URL].

## Declaration of interest's statement

The authors declare no conflict of interest.

## Appendix A Supplementary data

Supplementary Material_STOTEN Supplementary Material_STOTEN
