# Supplementary material for: Understanding the factors affecting the quantity and composition of street litter: Implication for management practices
Source: Heliyon. 2023 Mar 15;9(3):e14245. doi: 10.1016/j.heliyon.2023.e14245 (PMC10036511; doi:10.1016/j.heliyon.2023.e14245)
Supplement: Supplementary Material_STOTEN [file mmc1.docx]

**SUPPLEMENTARY MATERIAL**

**STOPPING TRASH WHERE IT STARTS - SITE DESCRIPTION**

1. Date ___________ Site ID_________ Name(s) of Surveyor(s) __________________________
2. Starts at: Lat____________ Long____________

Ends at: Lat____________ Long______________

1. Today: Temperature (°C/°F) _______ Rain (mm/inch) _________
2. Past 24 h: Avg Temp (°C/°F) _______ Rain (mm/inch) ________
3. Past 48 h: Avg Temp (°C/°F) _______ Rain (mm/inch) ________
4. Wind speed today (miles/h or km/h) __________________
5. Survey start time __________ Survey end time__________

1. Ground cover (%): Paved____ Grass_____ Shrubs/Bushes ____ Wooded _____Sand/Soil ________
2. Number of landscaped areas (e.g. flowers, mowed areas) ____________ Specify ___________
3. Number of people that walk by you:

1^st^ 10-min period ___________ 2^nd^ 10-min period __________ 3^rd^ 10-min period ___________

1. Number of vehicles that pass by you:

1^st^ 10-min period ___________ 2^nd^ 10-min period __________ 3^rd^ 10-min period _________

1. Total number of food-related business within the 800 x 800 m quadrant_______________
2. Number of Grocery stores (e.g. Shop Rite, Walmart, Trader Joe’s) _______________
3. Number of Convenience stores (e.g. 7-eleven, Dollar Tree)_____________________
4. Number of Restaurants/Diners (e.g. Olive Garden, IHOP) ______________________
5. Number of Coffee shops (e.g. Starbucks, Dunkin Donut) _______________________
6. Number of Fast-foods (e.g. Mc Donald’s, Burger King) _________________________
7. Number of Food carts (e.g. hot dog, halal, bagels) ____________________________
8. Number of Food trucks (e.g. Ice cream truck) ________________________________
9. Number of Other ___________________ Describe_______________________
10. Number of open bed vehicles (e.g. construction trucks, road maintenance) _________________
11. Number of Public areas within the block ___________ near-by________ Distance _____
12. Number of Construction sites within the block_______ near-by ________ Distance _____
13. Number of Loading docks within the block __________ near-by ________ Distance _____
14. Number of Public buildings within the block _________ near-by ________ Distance _____
15. Number of trash cans on the block (both sides of the street) _____________ (If 0 skip to question 21)
16. Number of Trashcan with plastic bag liner ______________ Without __________
17. 100% full______ 75% full_____ 50% full_____ 25% full______ 0% full_______
18. Number of trashcan with trash on the ground around the trashcan ______________
19. Among the trashcans with trash on the ground next to them, how many are:

100% full______ 75% full_____ 50% full_____ 25% full______ 0% full_______

1. Number of recycling bins on the block (both sides of the street) _________ (If 0 skip to question 21)
2. 100% full______ 75% full_____ 50% full_____ 25% full______ 0% full_______
3. Number of recycling bins with litter on the ground around the bin ______________
4. Among the recycling bins with litter on the ground next to them, how many are:

100% full______ 75% full_____ 50% full_____ 25% full______ 0% full_______

1. Number of manhole covers ____________________
2. Have you seen anyone collecting plastic bottles/cans? Yes ______ No _______
3. Number of storm drains (both sides of the street) ______________
4. Number of storm drains clogged with litter or debris:

100% clogged__ 75% clogged__ 50% clogged__ 25% clogged__ 0% clogged__?

1. Is there a particular spot in which you see the most litter (next to trash bins, on storm drains, on sidewalk, on the road, in tree pits, etc.)?_____________________________________________
2. Pictures taken before & after site collection (Y/N) __________________________________________

Notable/Unusual weather conditions (or NOTES in general) _____________________________________ __________________________________________________________________________________________________________________________________________________________________________

Measurements of the collected trash (provide at least one decimal place in each measurement): _____________________________________________________________________________________

1. Volume determination of the bin:
2. Bin sides (cm): *height*_________ (cm) *width*_________ (cm) *length*___________ (cm)
3. Volume of the bin: *height* x *width* x *length* = __________ (cm^3^)
4. Volume determination of the trash collected:

Trash volume (full bin) (Same volume of the bin at b): __________________________ cm^3^

Trash volume (not-full bin): ______________________________________________ cm^3^

- Measure the *new height* of the trash inside the bin
- As for the *length* and *width* use the bin’s measures from a).
- Compute the volume applying the multiplication at b).

1. Weight determination of the trash:

Put either the trash bag(s) or the bin with the garbage content on a digital scale.

If using the bin, weight the bin first or put the bin on the scale first and reset the scale to 0. Then weight the content, either loose or inside a trash bag.

Trash weight: _______________________________ kg (one decimal point minimum).

NOTE: If measures are taken in pounds or inches or feet, this MUST be specified.

| **Bin with trash – example of FULL bin** | **Bin with trash – example of a NOT-FULL bin** |
| --- | --- |
| 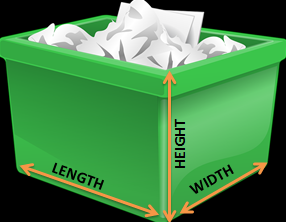 | 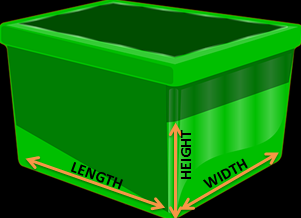 |

**STOPPING TRASH WHERE IT STARTS - TALLY FORM**

Site ID ________________ Date __________________

| Category | Subcategory | Material | Tally | Brand/Notes | |
| --- | --- | --- | --- | --- | --- |
| DRINKS CONTAINERS AND PARTS | Liquor Bottles | Glass |  |  | |
|  | Non-Liquor Bottles | Plastic |  |  | |
|  |  | Glass |  |  | |
|  |  | Metal |  |  | |
|  | Juice boxes | Composite |  |  | |
|  | Cups | Styrofoam |  |  | |
|  |  | Plastic |  |  | |
|  |  | Paper |  |  | |
|  |  | Glass/Ceramics |  |  | |
|  | Caps | Plastic |  |  | |
|  |  | Metal |  |  | |
|  | Lid | Plastic |  |  | |
|  |  | Metal |  |  | |
|  | Straw | Plastic |  |  | |
|  | Coffee stirrer | Plastic |  |  | |
|  | Cup sleeves | paper |  |  | |
|  | Four or Six pack rings for cans | Plastic |  |  | |
|  | Bottle neck ring | Plastic |  |  | |
|  | Liquor Cans | Metal |  |  | |
|  | Non-Liq. Cans | Metal |  |  | |
|  | Drink carrier/tray | Paper |  |  | |
|  | Pull tabs | Metal |  |  | |
| FOOD WRAPPING & PACKAGING | Gum/Snacks/ candies Wrappers | Plastic |  |  | |
|  |  | Aluminum |  |  | |
|  |  | Paper |  |  | |
|  | Utensils | Plastic |  |  | |
|  | Ziplock bag | Plastic |  |  | |
|  | Lollipop stick | Paper |  |  | |
|  |  | Plastic |  |  | |
|  | Popsicle stick | Plastic |  |  | |
|  |  | Wood |  |  | |
|  | Food Wrappers/Packaging | Plastic |  |  | |
|  |  | Styrofoam |  |  | |
|  |  | Metal |  |  | |
|  |  | Paper |  |  | |
|  |  | Composite |  |  | |
|  | Food Containers | Plastic |  |  | |
|  |  | Styrofoam |  |  | |
|  |  | Metal |  |  | |
|  |  | Paper |  |  | |
|  |  | Composite |  |  | |
|  | Plates | Styrofoam |  |  | |
|  |  | Paper |  |  | |
|  |  | Glass/Ceramics |  |  | |
|  |  | Metal |  |  | |
|  |  | Plastic |  |  | |
| MEDICAL RELATED | Drug vials | Plastic |  |  | |
|  | Drug vials with content | Composite |  |  | |
|  | Condoms | Plastic |  |  | |
|  | Bandages | Plastic |  |  | |
|  | Wound wrapping | Textile |  |  | |
|  | Syringe | Composite |  |  | |
|  | Pipette tips | Plastic |  |  | |
| ORGANIC WASTE | Human waste | Organic |  |  | |
|  | Loose Pet waste | Organic |  |  | |
|  | Wrapped Pet waste | Composite |  |  | |
|  | Food waste | Organic |  |  | |
|  | Yard waste | Organic |  |  | |
|  | Leaves | Organic |  |  | |
| LARGER AND OR HOUSEHOLD ITEMS | Furniture | Composite |  |  | |
|  | Mattresses | Composite |  |  | |
|  | Bags with trash | Composite |  |  | |
|  | Tires | Plastic |  |  | |
|  | Appliances | Metal |  |  | |
|  | Shopping carts | Metal |  |  | |
|  | Vehicle batteries | Composite |  |  | |
|  | Bike | Composite |  |  | |
|  | Bike wheel | Composite |  |  | |
|  | Vehicle wheel | Composite |  |  | |
|  | Vehicle (specify) | Composite |  |  | |
|  | Vehicle parts | Plastic |  |  | |
|  |  | Metal |  |  | |
| TOBACCO PRODUCTS | Lighters | Composite |  |  |  |
|  | Cigarette/cigars butts | Composite |  |  |  |
|  | Tobacco wrap (cellophane) | Plastic |  |  |  |
|  |  | Cellophane/  Foil |  |  |  |
|  | Tobacco box | Paper |  |  |  |
|  | Cigarette holder | Plastic |  |  |  |
|  | Matches | Composite |  |  |  |
| CONSTRUCTION MATERIALS/TOOLS | Concrete waste | Rock |  |  |  |
|  | Bricks | Rock |  |  |  |
|  | Wood boards | Organic |  |  |  |
|  | Wood chips | Organic |  |  |  |
|  | Rebar | Metal |  |  |  |
|  | Tiles | Rock |  |  |  |
|  | Tarp | Plastic |  |  |  |
|  | Tools | Composite |  |  |  |
|  | Gloves | Textile |  |  |  |
| MISCELLANEOUS | Balls (type) | Plastic |  |  |  |
|  | Toys | Plastic |  |  |  |
|  | Toys | Textile |  |  |  |
|  | Non-vehicle batteries | Composite |  |  |  |
|  | Pen/pencil | Plastic |  |  |  |
|  |  | Metal |  |  |  |
|  |  | Wood |  |  |  |
|  | Chemical containers | Composite |  |  |  |
|  | Personal care bottle | Plastic |  |  |  |
|  | Home care bottle | Plastic |  |  |  |
|  | Make up item | Plastic |  |  |  |
|  |  | Composite |  |  |  |
|  | Greasy layer on water (either oil or surfactant) | Composite |  |  |  |
|  | Spray paint cans (or bottles) | Composite |  |  |  |
|  | Hose/Pipe parts | Plastic |  |  |  |
|  |  | Metal |  |  |  |
|  | Wire/cable/rope | Plastic/  Synthetic |  |  |  |
|  |  | Metal |  |  |  |
|  |  | Electric |  |  |  |
|  |  | Composite |  |  |  |
|  | Tarp | Plastic |  |  |  |
|  | Foam materials | Styrofoam |  |  |  |
|  | Dryer sheets | Textile |  |  |  |
|  | Non-food Wrappers/Packaging | Plastic |  |  |  |
|  |  | Metal |  |  |  |
|  |  | Styrofoam |  |  |  |
|  | Human diapers/pads | Composite |  |  |  |
|  | Wipes | Textile |  |  |  |
|  | Tampon applicators | Plastic |  |  |  |
|  | Grocery/Shopping bags | Plastic |  |  |  |
|  |  | Textile |  |  |  |
|  |  | Paper |  |  |  |
|  | Non-food containers | Plastic |  |  |  |
|  |  | Metal |  |  |  |
|  |  | Styrofoam |  |  |  |
| MISCELLANEOUS | Product tag/label | Paper |  |  |  |
|  |  | Plastic |  |  |  |
|  |  | Textile |  |  |  |
|  |  | Metal |  |  |  |
|  | Newspaper | Paper |  |  |  |
|  | Magazine | Paper |  |  |  |
|  | Office paper | Paper |  |  |  |
|  | Cardboard | Paper |  |  |  |
|  | Tissue/Napkin | Paper |  |  |  |
|  | Flyer | Paper |  |  |  |
|  | Shoe/Boot | Composite |  |  |  |
|  | Clothes | Fabric |  |  |  |
|  | Bedding | Fabric |  |  |  |
|  | Cleaning bottles/spray | Plastic |  |  |  |
|  |  | Metal |  |  |  |
|  | Dead animals | Organic |  |  |  |
| FRAGMENTS | Fragments/  Pieces | Glass |  |  |  |
|  |  | Plastic |  |  |  |
|  |  | Textile |  |  |  |
|  |  | Paper |  |  |  |
|  |  | Metal |  |  |  |
|  |  | Styrofoam |  |  |  |
|  |  | Composite |  |  |  |
|  |  | Other |  |  |  |
| OTHERS (SPECIFY) |  |  |  |  |  |
|  |  |  |  |  |  |
|  |  |  |  |  |  |
|  |  |  |  |  |  |
|  |  |  |  |  |  |

### STOPPING TRASH WHERE IT STARTS – SURVEY PROTOCOL

**Survey dates:**

The survey will take place on pre-scheduled dates which will depend on the absence of major rain events as well as the absence of street sweeping in the previous 48 hours.

**Data forms and data insertion:**

Both the SURVEY FORM and the TALLY FORM will be converted into a fillable pdf file once the final versions will be official. The fillable pdf files will be uploaded into iPads and surveyors will insert the information collected on site in these fillable forms. The forms will be saved using the following labeling code: DATE_SITEID. (DATE should be in the format *ddmmyy*).

The Site ID of each location will be provided to the surveyors in a list of sites complete of street names delimiting the area to be surveyed and indicative coordinates (longitude and latitude). This list will be completed at the end of the sites selection and scouting evaluation. The SURVEY FORM is the first one that has to be completed. After completion of this document, the surveyor can move to the TALLY FORM, qualifying and quantifying the trash items found.

**Meteorological information:**

Air temperature (T) and rain during survey, average diurnal air T and cumulative rain in the previous 24 h, average diurnal air T and cumulative rain in the previous 48 h, and wind speed during the survey, should be noted from reliable sites on the same day, before heading to the site (e.g. NOAA, Newark International Airport).

**Survey start time and survey end time:**

They should be written in military format (24 hour-method). Example: instead of 2 pm it should be noted as 14:00. In this way, AM and PM specifications are not needed.

**Names of the surveyors:**

Complete first and last names of the surveyors are needed. Moreover, the initials of each surveyor should be reported in parentheses next to the full name.

Assessment parameter table:

This table is meant to provide an initial general visual assessment of the visited site. Since this information is mostly subjective to the surveyor’s opinion, both surveyors at each site should select the value that better describes (from least disturbed to most disturbed) each of the four parameters provided in the assessment parameter table. Two lines are provided in the table (one per each surveyor). No need to indicate who is surveyor 1 or 2. The values assigned to each parameter under evaluation will be averaged and each averaged value will be summed up to a final score reported out of the total of 80 possible points. This final score will help us to classify each site based upon an initial visual evaluation. For each parameter, four situations are described, and for each situation 5 values are available.

1. Trash level first glance: the surveyors should express in a value from 0 to 20, what is the level of trash presence in a first glance (pictures with examples to be provided).
2. Access to the waterbody from the site: each site was selected at a maximum distance of 300 m from the waterbody meaning that they are located fairly close to the waterbody. Despite their vicinity, some site may not have direct access to the waterbody (e.g. there is a private passage and/or a gate is present; it is densely vegetated and no pathways are cutting through the vegetation). On the other hand, direct access could be present that would easily allow for trash to accumulate along the shoreline.
3. Floatability of litter found: the amount of trash found at the site might be significant but only a part of it is light enough to be easily transported by the wind or surface runoff to the waterbody. Estimate the approximate quantity of items that potentially could reach the water because of light-weighted items (e.g. plastic, Styrofoam, paper, cardboard). This value can be assigned after the tally procedure has been completed. In this way, the surveyors will have a better idea of the real quantity of floatable items present on the street.
4. Large or household items: in addition to the light trash that potentially could be transported to the waterbody, evaluate the eventual presence of large and/or heavy items or any household object dumped on the street illegally that would negatively affect aesthetics.

Ground cover (%): The surveyor is required to estimate the percent coverage at the ground level of the following ground cover categories: 1) Paved 2) Grass 3) Shrubs/Bushes 4) Wooded 5) Sand/Soil. For instance: Paved 80%, Grass 0%, Shrubs/Bushes 5%, Wooded 5%, and Sand/Soil 10%.

Landscaped areas: Usually, signs of beautification like, presence of flowers, mowed areas, trimmed bushes, is a sign of people taking care of the neighborhood and interested in keeping the area clean. Regarding this, remember not to collect any trash from front yards along the segment to be surveyed. We are conducting a survey, we are not provide a service.

People and vehicles that pass by you**:** This information will provide an idea of how busy the site is and the relative frequentation of vehicles versus pedestrians. While two surveyors are collecting and tallying the trash found along the surveyed segment, a third surveyor will be in charge of counting people and vehicles passing by in the street segment designated for the survey. The surveyor will conduct the count for 10 consecutive minutes using tally counters. One tally counter will be used for counting the vehicles and one for counting pedestrians. Ideally, the 10-minute intervals should be repeated two more times any time during the permanence of the surveyors on the site that date.

Food related business activities within the 800 x 800 m quadrant**:** Distinguish the different types of food related businesses with the purpose of later finding the sources of litter, especially when the wraps and containers found are showing a brand. The different types are grocery and convenience stores, restaurants are grouped with diners, coffee shops, fast-foods. These are the stores that are always present, but there also could be food-carrying vehicles like ice cream trucks and carts. Some examples are provided in parentheses for each category and an extra line is available for any other food business not listed.

Open bed vehicles: Vehicles that do not cover or secure their loads may allow for the release of items into the environment while operating or when parked and this may represent a noticeable contribution of trash to local waterways and also to Municipal Separate Storm Sewer System (MS4s). New Jersey regulates this issue through section 39:4-77 and any violator may be fined. For this reason, surveyors should write notes (e.g. plate number, construction business name) of any open-bed truck vehicle, within the surveyed segment site, which load is not secured.

Public areas, constructions sites, loading docks, public buildings: These areas are potential sources or carriers of trash. Public areas include playgrounds and parks and may be a source of food and drink-related containers and packages. Construction sites and loading docks may be sources of big plastic wraps, debris and cardboard. Public buildings like hospitals, libraries, and post offices might represent highly-frequented meeting areas that may result in litter generation. These trash sources might be within the delineated surveyed area (within the 400m pre-determined segment(s)) and/or near-by. In this last case, the surveyors have to estimate the approximate distance (in m) from the limits of the surveyed block.

Trash cans and recycling bins: The presence of trash cans and recycling bins is extremely important in the intent to keep the environment in which we live clean. The higher the number of these containers and the better they are maintained to keep the street clean. In addition, the presence or absence of plastic bag liners is important especially when liquids are disposed and when trash is in small pieces. The presence of a liner would prevent liquids and small parts to be spread out on the ground and also leach into draining systems when rain dilutes and washes them. It is important to be consistent in tallying the bins on both sides of the segment visited and consistency should be applied to all sites. IMPORTANT: Trash bins have to be counted on both sides of the investigated segment.

Manhole covers: A manhole cover is a small opening in the street and covered by a lid, in order to allow staff in charge of maintenance to have access underneath it. This opening usually leads to a sewer. Depending on how the cover is made and what condition it is in, there could be a passage for trash. IMPORTANT: Manholes have to be counted on both sides of the investigated segment.

Collectors of recyclables: At times, people are seen sorting plastic bottles and aluminum cans from the trash bins and from the street and collecting them in big plastic bags to sell to recycle points. If one of these individuals is seen, it is important to record it on the notes section because they may remove these items in that area and cause a bias in the tallying. Please include in your notes the number of people seen actively collecting or sorting through plastic bottles and aluminum cans.

Storm drains: Storm drains in between the curb and the street usually have large openings to allow a good drainage of stormwater flow along the street. Unfortunately, when trash is present on the street, it can be transported along with the same stormwater and very often ends in these storm drains. Items smaller than the openings can be drained together with the stormwater but larger items may remain stuck against the storm drain structure. IMPORTANT: Stormdrains have to be counted on both sides of the investigated segment.


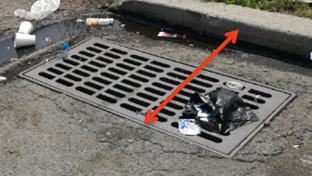


Particular spot with a lot of litter: Surveyors should write down (and take a picture) if they see any particular spot in which the debris seems to accumulate most. The location of accumulated debris could shed light to the movement of material at that site or about particular activities or conditions which should receive the most attention.

Pictures: Suggest taking pictures of significant areas/points/events/situations. Before doing this, the first picture to take should be of a white board showing site-specific information (date, time, Site ID) written with a dry erasable marker. In this way all the following pictures taken at the site will be consecutive to the board displaying the site-specific information.

General Notes: Surveyors can write here anything that they think might need to be mentioned. For example if there is any unusual weather condition or activity.

Measurements of the collected trash:

Volume determination of the bin (cm^3^): the measurements of the bin should be taken and multiplied by one another. This will provide the volume of the bin.

Volume determination of the trash collected (cm^3^): this value will be the same of the volume of the bin if the bin is filled with trash. If the bin is not full of trash, only the height of the bin will be different. The surveyor will have to measure the new height and multiply this new measurement by the same width and length. If both a full bin AND a portion of the bin are the case, both values need to be reported in the proper blank spaces.

Weight determination of the trash (kg): the surveyor should put the trash on a field scale and record it in the proper blank spaces (one decimal point). Put either the trash bag(s) or the bin with the garbage content on a digital scale. If using the bin, weight the empty bin first or put the bin on the scale first and reset the scale to 0. Then weight the content, either loose or inside a trash bag. NOTE: If measures are taken in pounds or inches or feet, this MUST be specified.

Detailed instructions regarding how to determine the volume and the weight of the trash are provided in the SITE DESCRIPTION FORM (Appendix 1). The purpose of recording both volume and weight of the collected trash at the different locations is to provide results that can be compared with other projects even when the sites are in a totally different area. In some projects information about trash surveys is reported as count of items. In other researches, weight of the trash collected is provided. Finally, trash may be reported as a volume estimation. Having the opportunity to record both counts, weight and volume of the litter will provided an exhaustive set of information which will allow different projects with different methodologies applied to be compared.

## Tally Form Protocol:

The first column lists the ten categories we grouped the item into: Drink containers and parts, Food wrapping and packaging, medical related, Organic waste, Larger and/or household items, Tobacco products, Construction material /tools, Miscellaneous, Fragments, Others.

The second column shows a long list of subcategories per each category, describing in details the several items that might be found as trash in an area. The subcategories indicate the individual items that will be tallied.

In the third column are listed all possible materials the individual items can be made of. The different materials listed are: metal, plastic, paper, glass, composite (when more than one material is present in the same item), Styrofoam, textile, fabric, organic (e.g.: food waste, material from pruning), and rock (e.g.: concrete, brick, tile).

The fourth column is for the tally. The person in charge tally lines (|) for each subcategory in the specific material it has been found. Tallies will be added up during the data analysis phase. An ordinal number can also be reported for the final count of the items when, for instance, counting the individual items piled up for each group of subcategory. A trash grabber or metal tongs and or latex/textile gloves should be used for picking up the trash.

The last column is intended for notes of any type. In particular, whenever it is possible and clear, the brand of the tallied item clearly coming from a particular store/discount/retailer should be specified. This will later help to trace back the sources of particular trash items and evaluate what could be done to reduce these sources.

Once trash has been tallied it has to be disposed in trash or recycle bags (except the large and/or heavy items) in plastic bags.
